# Supplementary material for: Empathi: embedding-based phage protein annotation tool by hierarchical assignment
Source: Nat Commun. 2025 Oct 14;16:9114. doi: 10.1038/s41467-025-64177-5 (PMC12521583; doi:10.1038/s41467-025-64177-5)
Supplement: Supplementary file 1 — Supplementary Information [file 41467_2025_64177_MOESM1_ESM.pdf]

Empathi: Embedding-based Phage Protein Annotation Tool by Hierarchical Assignment

Alexandre Boulay, Audrey Leprince, François Enault, Elsa Rousseau and Clovis Galiez

Supplementary Information

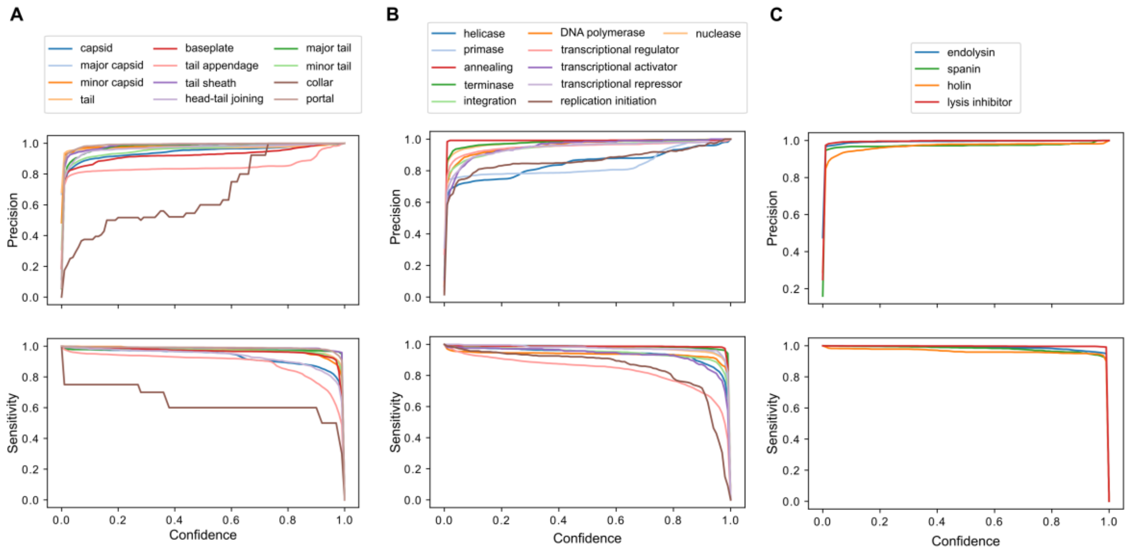

Supplementary Figure 1. Precision and sensitivity curves as a function of confidence for Empathi models trained on **A)** subcategories of structural proteins, **B)** subcategories of DNA-associated proteins and **C)** subcategories of lysis-associated proteins.

Supplementary Table 1. Number of proteins and protein clusters used to train and test Empathi models as well as performance metrics obtained on the test set.

	Training				Testing				Empathi testing scores		
	# Pos clusters	# Pos proteins	# Neg clusters	# Neg proteins	# Pos clusters	# Pos proteins	# Neg clusters	# Neg proteins	F1-score	Precision	Recall
**Packaging and assembly**	2723	26373	36668	36668	666	6338	9182	9182	0.97	0.978	0.95
**PVP**	12971	108029	26404	26404	3219	28080	6625	6625	0.98	0.99	0.96
**Tail**	8304	67259	2640	28392	2086	15886	650	7422	0.98	0.97	0.98
**Major tail**	428	5324	6373	53817	94	647	1607	11002	0.98	0.99	0.97
**Minor tail**	1363	16107	5408	39877	346	4260	1347	9507	0.97	0.97	0.98
**Capsid**	1219	12471	9734	82447	282	2828	2457	21446	0.96	0.96	0.96
**Major capsid**	510	7616	559	4090	145	1625	123	805	0.98	0.98	0.99
**Minor capsid**	487	3337	581	8597	118	1054	150	1126	0.98	0.98	0.97
**Baseplate**	964	11108	5825	46684	251	2403	1447	10542	0.95	0.93	0.97
**Tail appendage**	2647	12798	3959	41375	644	3639	1008	10989	0.88	0.84	0.92
**Portal**	520	6711	10458	85116	130	1669	2615	26237	0.99	1	0.99
**Collar**	31	213	10943	95230	8	20	2736	24161	0.6	0.6	0.6
**Tail sheath**	222	2458	6579	54462	59	851	1642	13029	0.99	1	0.99
**Head-tail joining**	717	8860	10257	89311	173	1397	2571	20126	0.97	0.98	0.96
**DNA-associated**	12581	105873	24857	24857	3106	27216	6254	6254	0.98	0.99	0.96
**Integration**	1792	14657	9876	84294	465	3192	2453	23266	0.96	0.97	0.96
**Nuclease**	3406	27434	8254	72908	857	6890	2059	18200	0.98	0.99	0.99
**DNA polymerase**	717	8799	10843	90200	180	1817	2710	23414	0.96	0.98	0.94
**Terminase**	1124	11456	10569	88568	255	3374	2669	23646	0.99	0.99	0.99
**Annealing**	275	2757	11431	97824	68	877	2859	25471	0.99	0.99	0.99
**Helicase**	811	9913	10714	90371	182	1579	2700	21902	0.91	0.87	0.96
**Primase**	345	3584	11215	94299	69	1182	2822	25432	0.88	0.8	0.99
**Replication initiation**	395	1642	11289	100722	83	377	2839	24146	0.89	0.86	0.92
**Transcriptional regulator**	3183	14695	35429	35429	776	3307	8877	8877	0.91	0.96	0.86
**Transcriptional activator**	158	924	1530	7401	40	209	382	1696	0.97	0.99	0.95
**Transcriptional repressor**	842	3057	846	5521	200	515	222	1137	0.97	0.97	0.98
**Adsorption-related**	2581	12561	36703	36703	671	3558	9150	9150	0.93	0.92	0.94
**RNA-associated**	892	9347	38580	38580	257	2820	9612	9612	0.94	0.99	0.9
**Nucleotide metabolism**	920	12696	38556	38556	240	2898	9629	9629	0.98	0.99	0.98
**Phosphorylation**	1057	11182	38408	38408	245	1676	9622	9622	0.95	0.97	0.94
**Transferase**	1621	13721	37846	37846	385	2517	9482	9482	0.96	0.98	0.94
**Reductase**	455	6881	39043	39043	121	1851	9754	9754	0.98	1	0.97
**Ejection**	438	4364	38265	38265	99	655	9577	9577	0.93	0.94	0.92
**Cell wall depolymerase**	2038	19677	37373	37373	482	4003	9371	9371	0.93	0.98	0.89
**Anti-restriction**	964	6580	38503	38503	228	1825	9639	9639	0.96	0.99	0.93
**Crispr**	44	269	39453	39453	6	149	9869	9869	0.98	0.98	0.97
**Sir2**	39	549	39461	39461	4	39	9872	9872	1	1	1
**Super infection**	163	817	39315	39315	45	376	9825	9825	0.95	0.96	0.94
**Toxin**	271	1013	39189	39189	59	280	9806	9806	0.9	0.98	0.83
**Lysis**	2030	18289	37418	37418	469	5286	9394	9394	0.96	0.98	0.94
**Endolysin**	895	9140	1102	9239	242	2412	258	2658	0.99	1	0.99
**Holin**	561	4163	1436	14453	143	1396	357	3437	0.97	0.98	0.96
**Spanin**	383	2923	1616	15422	111	839	389	4391	0.98	0.97	0.99
**Lysis inhibitor**	146	1364	1853	17038	44	1290	456	3883	1	1	1

Supplementary Table 2. Proteins in the PP079085.1 genome from Ni et al. study for which the Empathi prediction differs from the PHROG category but is coherent with the more precise PHROG annotation.

Position in genome	Empathi	PHROG annotation	PHROG category	PHROG entry	VPF-PLM
1	Lysis	Endolysin	Head and packaging	phrog_2860	Head and packaging
8	Internal/ejection	Internal virion protein	Head and packaging	phrog_308	Head and packaging
11	Internal/ejection	Internal virion protein	Head and packaging	phrog_418	Head and packaging
12	Internal/ejection	Internal virion protein	Head and packaging	phrog_6651	Head and packaging
17	Transferase	Glycosyltransferase	Moron, auxiliary metabolic gene and host takeover	phrog_2	Head and packaging
18	Transferase	Glycosyltransferase	Moron, auxiliary metabolic gene and host takeover	phrog_11494	Head and packaging
28	DNA-associated<br><br>Packaging/assembly	Terminase large subunit	Head and packaging	phrog_14945	Unknown
29	DNA-associated<br>Packaging/assembly	Terminase small subunit	Head and packaging	phrog_34859	Moron, AMG and host takeover
46	Lysis	Endolysin	Head and packaging	phrog_2860	Unknown

Supplementary Note 1

In order to evaluate Empathi on proteins that are “out-of-distribution” compared to the phage proteins used to train Empathi, 500 proteins were randomly selected from SwissProt[1]. These are mostly non-phage proteins and are consequently not expected to be assigned phage-specific functions. Indeed, 287/500 were unassigned by Empathi and many (129) only had high-level functional annotations (e.g. DNA/RNA associated, transferase, transcriptional regulator, etc.). More specific annotations were also assigned to some proteins, for example, 15 integration-related proteins and 10 nucleases were assigned by Empathi.

In regard to proteins that received phage specific annotations, 26/500 were annotated as phage virion proteins (PVPs). Most have low-moderate confidence (<80%) but presented here are the ones predicted with high confidence:

- Q45753 has a confidence of 96%. Albeit not being a PVP, it is a protein containing a galactose-binding domain (see Family and domains tab). This domain (IPR008979) is often found in phage tail proteins and receptor binding proteins (see the BFVD[2] tab on the InterPro[3] page) for phages to adsorb to the bacterial cell wall surface, and likely to carbohydrates containing, among others, galactose[4].
- C0HJH8 is a major capsid protein predicted with 99.7% confidence.
- A1V7N8 (confidence 90%) is a flagellar hook-basal body complex protein, so once again not a PVP, but a protein composing the structure of the flagellum of bacteria and acting as a connector.
- P0DOS2 (confidence 84%) plays a part in the formation of the virosome of the variola virus.
- P0C2W1 (confidence 82%) is a multifunctional protein found in many biological processes. The IPR013320 domain is found in tail fiber proteins and minor tail proteins (see the BFVD tab on the InterPro page).
- Q84GK0 (confidence 82%) possesses many tailspike related domains including IPR012332 (pectate lyase) and IPR009003 (peptidase).

To summarise, although most of these proteins are false positives (i.e. they may not be PVPs), they share domains with PVPs and if they were found in phages, might adopt these roles.

Next,13/500 were predicted as being cell wall depolymerases (degradation of sugars and peptides). Again, focusing only on high-confidence (i.e. above 80%) predictions:

- Q8DTC7 had a confidence of 95%. It is a chaperone and protease (PTHR11638). It is not a depolymerase, but chaperones are often part of tail fibers which assemble in trimeric structures to help with assembly[5].
- Q2YSD6 (confidence 86%) possesses the same protease domain as the previous protein.
- A0A1G9FQX8 (confidence 87%) is an adenosyl transferase and hydrolase. It was also predicted as being a transferase with 87% confidence.
- Q8AY81 (confidence 84%) has endopeptidase activity (IPR009003).

- Q2FWJ6 (confidence 96%). Contains the sprt-like domain (IPR006640), a protease found in autolysins of S. pneumoniae[6].

All in all, this test, performed using proteins mostly unrelated to phages, shows that Empathi has a low false positive rate on proteins that are distinct from those seen during training. Additionally, most false positives identified possess domains usually found in phage tail proteins or depolymerases.

Supplementary References

1. Boutet, E., Lieberherr, D., Tognolli, M., Schneider, M. & Bairoch, A. UniProtKB/Swiss-Prot. in *Plant Bioinformatics: Methods and Protocols* (ed. Edwards, D.) 89–112 (Humana Press, Totowa, NJ, 2007). doi:10.1007/978-1-59745-535-0_4.
2. Kim, R. S., Levy Karin, E., Mirdita, M., Chikhi, R. & Steinegger, M. BFVD—a large repository of predicted viral protein structures. *Nucleic Acids Res* **53**, D340–D347 (2025).
3. Blum, M. *et al.* InterPro: the protein sequence classification resource in 2025. *Nucleic Acids Res* **53**, D444–D456 (2025).
4. Tremblay, D. *et al.* Receptor-Binding Protein of Lactococcus lactis Phages: Identification and Characterization of the Saccharide Receptor-Binding Site. *Journal of Bacteriology* **188**, (2006).
5. North, O. I. & Davidson, A. R. Phage Proteins Required for Tail Fiber Assembly Also Bind Specifically to the Surface of Host Bacterial Strains. *Journal of Bacteriology* **203**, 10.1128/jb.00406-20 (2021).
6. Marquart, M. E. Pathogenicity and virulence of Streptococcus pneumoniae: Cutting to the chase on proteases. *Virulence* **12**, 766 (2021).
